# The Real-World Evaluation of Remote Electrical Neuromodulation in Pediatric Migraines: A Preliminary Study

**DOI:** 10.3390/children12111500

**Published:** 2025-11-05

**Authors:** Amit Blumovich, Trevor Gerson, Mark Connelly, Tammie Wingert, Gina Jones

**Affiliations:** 1 Headache Clinic, Division of Pediatric Neurology, Children’s Mercy Hospital, Kansas City, MO 64108, USA; tgerson@cmh.edu (T.G.); mconnelly1@cmh.edu (M.C.); twingert@cmh.edu (T.W.); gljones2@cmh.edu (G.J.); 2 Sackler Faculty of Medicine, Tel Aviv University, Tel Aviv 6997801, Israel

**Keywords:** pediatric headache, migraine, remote electrical neuromodulation, Nerivio, acute treatment, non-pharmacologic therapy, real-world evidence

## Abstract

**Background/Objectives:** Pediatric migraine disrupts school performance and daily functioning. Concerns about medication overuse and limited efficacy highlight the need for non-pharmacologic treatments. The Nerivio remote electrical neuromodulation (REN) device, which is FDA-cleared for ages 8 and above, was evaluated in this study to assess real-world perceptions among patients in a pediatric neurology clinic. **Methods:** Patients aged 10–18 years who had used both acute medications and Nerivio completed two structured questionnaires, one reflecting on experiences with acute medication and one reflecting on experiences with acute REN treatment, assessing school and daily functioning, headache control, medication use, satisfaction, and preference. Descriptive statistics summarized the responses. **Results:** Twenty-four patients participated (91.7% female, mostly aged 13–18 years). Primary outcomes: Nerivio stopped headaches in 33.3% of patients and shortened them in 50.0%, with 41.7% reporting reduced medication use. Exploratory functional outcomes: Missed full school days were unchanged (3.8), partial absences decreased slightly (3.1 to 3.0, ~3%), limited-activity days declined from 3.5 to 2.7 (23%), and days with <50% functioning fell from 4.1 to 3.2 (22%). Preference favored Nerivio in 37.5%, medications in 20.8%, and both equally in 41.7%. Most patients (83.3%) wished to continue; 12.5% reported only mild, transient discomfort, and all continued treatment. **Conclusions:** This preliminary real-world study suggests that REN is feasible and beneficial in pediatric headache care. Primary outcomes demonstrated meaningful headache improvement, while exploratory measures suggested functional gains. REN reduced acute medication use and achieved high satisfaction, supporting its potential role as a patient-centered adjunct in pediatric headache management. Larger studies are needed to confirm these findings.

## 1. Introduction

Migraine and recurrent headache disorders are common in children and adolescents and may substantially interfere with their daily functioning, school performance, and quality of life [1]. Global migraine prevalence estimates reach approximately 8%, underscoring the importance of recognizing the disorder during formative developmental years [2]. In pediatric populations, acute treatment has traditionally relied on over-the-counter (OTC) medications, such as ibuprofen and acetaminophen, which have shown moderate efficacy in randomized trials of children aged ≥ 4 years, and their use is supported by international guidelines [3,4]. However, the frequent use of acute migraine medications can contribute to the development of medication overuse headache (MOH) [5], and treatment outcomes are variable. No single therapy for migraine has been shown to be clearly superior, and many patients require tailored interventions when pharmacologic therapy alone is insufficient [6].

Non-pharmacologic approaches, including neuromodulation, are increasingly recognized as promising adjuncts or alternatives for pediatric migraine. The Nerivio device is a remote electrical neuromodulation (REN) wearable that delivers 45 min of controlled electrical stimulation to Aδ and C nerve fibers in the upper arm when a migraine begins, activating conditioned pain modulation pathways that inhibit nociceptive signaling [7,8]. It was initially cleared by the FDA for acute migraine in individuals aged 12 years and older, with more recent clearance extending to children aged 8 years and above. Although pediatric evidence remains limited, prior studies have found that up to 60% of users experience pain relief within two hours, with improvements in functional outcomes and minimal adverse effects [7,8].

While these early studies support the safety and efficacy of REN, less is known about its real-world role alongside standard acute medications in pediatric practice, where treatment decisions are strongly influenced by patient and family experiences. Understanding these real-world, patient-centered perspectives is critical for guiding the adoption and integration of REN into routine migraine management. To address this gap, we undertook a preliminary real-world study aimed at characterizing the clinical and experiential outcomes of Nerivio.

## 2. Materials and Methods

This project was conducted at the Pediatric Neurology Clinic of Children’s Mercy Hospital, a tertiary care center in Kansas City, MO, USA, from 3 March to 30 June 2025. Eligible participants were children and adolescents aged 10–18 years with a clinical diagnosis of migraine, according to the International Classification of Headache Disorders (ICHD-3) criteria, who were using both over-the-counter (OTC) acute medications and the Nerivio device (Theranica Bio-Electronics Ltd., Netanya, Israel) for migraine treatment. Patients were recruited during routine neurology follow-up visits. All participants had previously used over-the-counter (OTC) acute medications before initiating Nerivio and were maintained on stable preventive regimens throughout the study period. The questionnaires were completed at a single timepoint, based on participants’ retrospective reports after they had gained experience with both treatments, ensuring consistent recall.

Data were collected using two structured questionnaires. The “acute medications” questionnaire assessed headache characteristics, school absences, functional disability, medication response, and satisfaction with acute pharmacologic therapy. The “acute treatment with REN” questionnaire evaluated perceived changes in headache burden, school attendance, activity limitation, medication use, and treatment satisfaction. In the REN questionnaire, patients were also asked to describe their perceived treatment effects using predefined categories: “stopped headache” (complete resolution of pain within two hours after stimulation), “shortened headache” (≥50% reduction in attack duration compared with typical episodes), and “less medication” (a subjective patient-reported decrease in the frequency or amount of acute medication used after starting Nerivio).

Information on the number of acute Nerivio treatments per participant was not systematically recorded; therefore, findings primarily reflect general real-world impressions following limited exposure to REN.

Questionnaires were initially distributed electronically through a REDCap-based clinical registry (Research Electronic Data Capture; Vanderbilt University, Nashville, TN, USA); however, due to low response rates, subsequent data were collected on paper during clinic visits. Participation was voluntary, and responses were anonymized.

Functional outcomes, including missed school days, reduced activity, and impaired functioning, were adapted from the Pediatric Migraine Disability Assessment (PedMIDAS), a validated instrument for measuring headache-related disability in pediatric populations. Because both questionnaires were completed concurrently for patients with prior documented use of both treatments, PedMIDAS items were used descriptively rather than as longitudinal measures. To align with the retrospective design, participants were asked to report functioning over a recent representative period rather than the original three-month PedMIDAS timeframe.

Descriptive statistics were used to summarize categorical responses and to estimate mean values for ordinal ranges (e.g., 1–2 days, 3–5 days). Results are presented as absolute values, mean changes, and percent reductions, which serve as effect size estimates to illustrate the magnitude and direction of change. Comparisons between baseline and post-Nerivio periods were descriptive; no formal inferential statistics were applied given the exploratory design and limited sample size. Data were visualized using bar charts and comparative plots.

## 3. Results

### 3.1. Study Population

**Figure 1:**
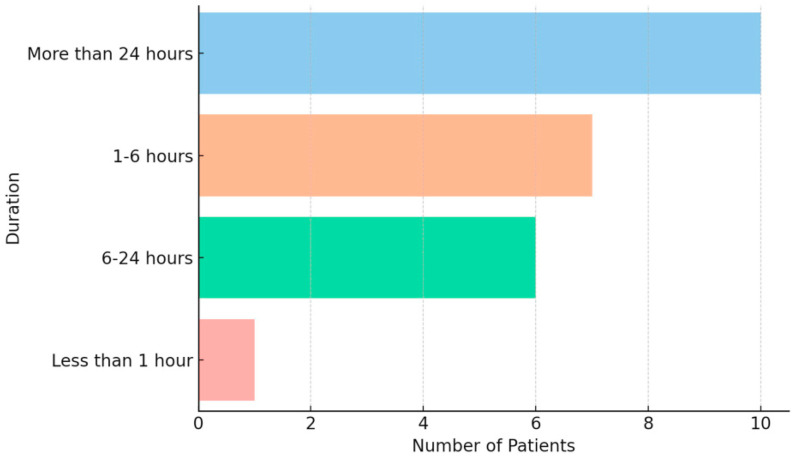
Distribution of headache episode duration reported prior to Nerivio use.

A total of 24 pediatric patients diagnosed with migraine according to the International Classification of Headache Disorders (ICHD-3), aged 10–18 years, were included. Most (87.5%) were in the 13–18-year-old age group, and 91.7% identified as female. This distribution reflects the well-documented higher prevalence of migraine in female adolescents and the randomized nature of the questionnaire distribution within the clinic. All participants reported a long-standing history of migraine attacks, typically lasting either 1–6 h or more than 24 h (Figure 1).

### 3.2. Treatment Effectiveness and Medication Reduction

**Figure 2:**
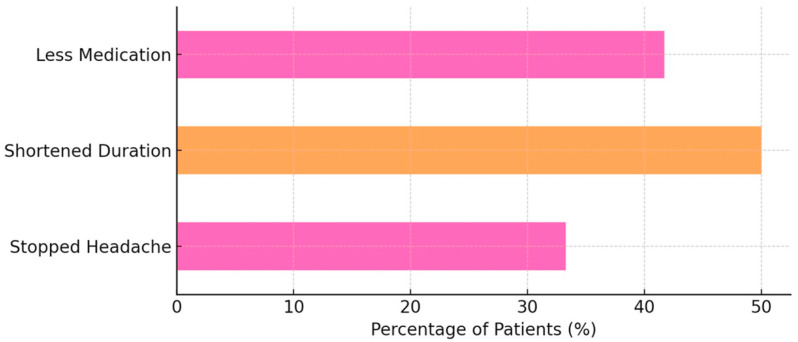
Percentage of patients reporting clinical benefit from Nerivio use.

When asked about the specific effects of Nerivio, 8 out of 24 patients (33.3%) reported that it “stopped the headache”. A further 12 out of 24 respondents (50.0%) indicated that it “shortened the headache,” and 10 out of 24 participants (41.7%) reported a reduced need for acute medications after Nerivio use (Figure 2).

### 3.3. Functional Outcomes Before and After Nerivio Use

**Figure 3:**
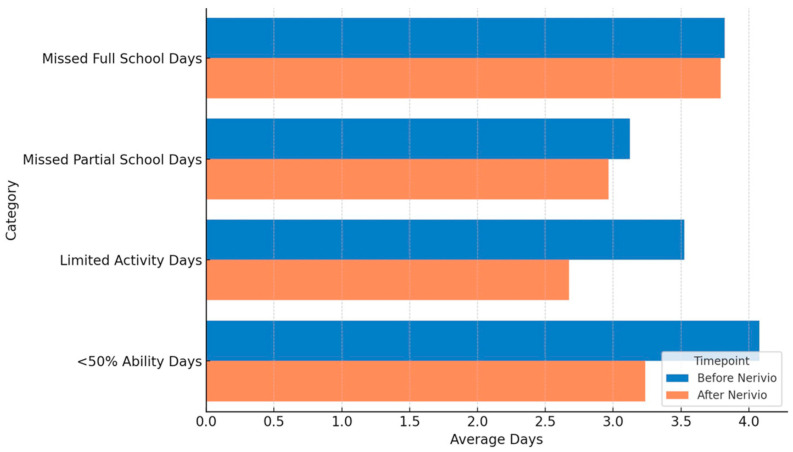
Mean number of days with missed school, reduced activity, and impaired function before vs. after Nerivio treatment.

Functional limitations due to headache were evaluated based on responses to the acute medication and REN questionnaires, reflecting retrospective reports of patients who had used both treatments. This design captured real-world perceptions of acute effects rather than preventive outcomes. Accordingly, functional data were summarized descriptively rather than interpreted as preventive effects. The average number of missed full school days remained stable at 3.8 days. Missed partial school days decreased slightly from 3.1 to 3.0 days (~3% reduction). Days for which other activities (e.g., play, sports) were stopped declined from 3.5 to 2.7 (23% reduction), and days with functioning at <50% ability decreased from 4.1 to 3.2 (22% reduction). Formal statistical testing was not performed given the sample size and preliminary design (Figure 3).

### 3.4. Treatment Preference

**Figure 4:**
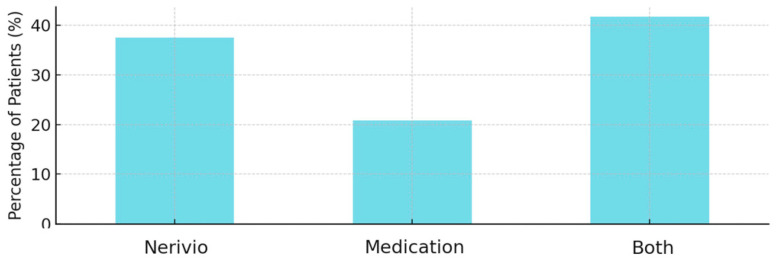
Patient preference for Nerivio vs. medications.

Treatment preference responses favored Nerivio in 9 of 24 patients (37.5%) and favored medications in 5 of 24 cases (20.8%). Patients demonstrated an equal preference for both treatments in 10 out of 24 cases (41.7%) (Figure 4).

### 3.5. Satisfaction and Tolerability

**Figure 5:**
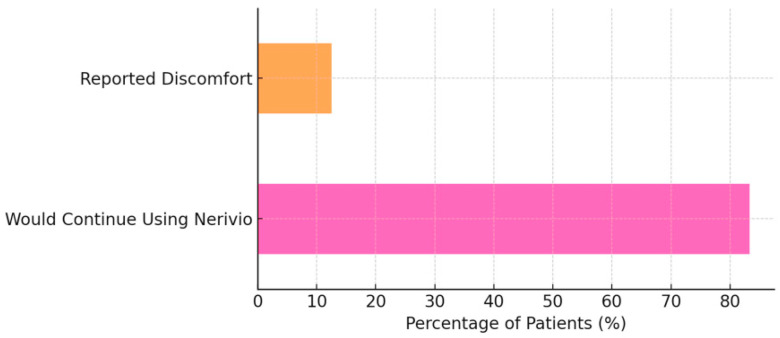
Percentage of patients reporting satisfaction and discomfort with Nerivio.

Most patients (20/24, 83.3%) expressed a willingness to continue using Nerivio. Three patients (12.5%) reported mild discomfort (e.g., local tingling or tightness at the stimulation site). All participants continued treatment despite these discomforts, which were consistent with expected device-related sensations (Figure 5).

## 4. Discussion

This preliminary study evaluated real-world patient experiences with Nerivio, a wearable remote electrical neuromodulation (REN) device, in the context of routine pediatric headache management. Consistent with prior clinical and real-world studies of REN [7,8], our findings provide preliminary evidence on functional outcomes, treatment satisfaction, and patient preferences in children and adolescents with migraine.

Although the average number of missed full school days in our cohort remained unchanged, Nerivio use was associated with fewer partial absences and a reduction in days with limited activity and <50% functioning. These exploratory functional findings parallel prior REN studies demonstrating decreased functional disability during migraine attacks [7,8] and align with broader evidence that non-pharmacologic interventions—including cognitive behavioral therapy, which has been shown to reduce headache days and migraine-related disability in children [9], and psychologically based approaches such as relaxation and biofeedback [10]—can support daily functioning and school attendance in pediatric populations with migraine.

As the primary outcomes, a large proportion of patients reported meaningful headache benefits with REN, including complete cessation in one-third and shortened durations in about one-half of participants. These findings also align with clinical trial data showing 58.9–74.2% pain relief and 20.0–35.6% pain freedom at 2 h [7], as well as adolescent trial findings of 71% pain relief and 35% pain freedom [8]. Importantly, 41.7% of patients required less acute medication after using Nerivio, supporting the potential of REN as a medication-sparing strategy [7,8]. This is particularly relevant in pediatric populations, where medication overuse headache, tolerability issues, and caregiver concerns remain persistent challenges [5,6].

Treatment preference results in our cohort varied, with a little over one-third favoring Nerivio, one-fifth favoring medication, and the remainder reporting equal benefits. This heterogeneity underscores the individualized nature of migraine care and reinforces the importance of offering a range of therapeutic options tailored to patient and family priorities. Nerivio tolerability was favorable, with only 12.5% of patients reporting discomfort and the majority (83.3%) expressing a willingness to continue use. Reported discomfort was mild and transient, described mainly as brief tingling or tightness at the application site, and all participants continued the treatment despite these sensations. These findings are consistent with prior REN safety data [7,8] and support the device’s integration as a non-pharmacologic adjunct in clinical practice.

Several limitations should be noted. The small sample size, single-site design, and reliance on self-reported outcomes limit generalizability and introduce a potential reporting bias. Because both acute medications and acute treatment with REN questionnaires were completed during the same visit, this study reflects retrospective perceptions rather than longitudinal outcomes, and therefore preventive or causal effects cannot be inferred. Detailed frequency data on headache episodes and acute medication use before and after Nerivio initiation were not systematically collected, as the study emphasized patients’ perceived experiences and real-world impressions of treatment efficacy. Additionally, some participants may have continued using acute medications alongside REN, which could partially confound treatment attributions. Finally, analyses were descriptive and exploratory, aimed at illustrating effect size trends rather than testing statistical significance.

Despite these limitations, this study contributes to the body of growing evidence that demonstrates that REN is feasible, acceptable, and potentially beneficial in pediatric migraine care. It highlights the importance of incorporating patient-reported experiences into evaluations of new therapies and suggests that REN may reduce functional disability and reliance on acute medications in everyday practice. Future research with larger, more diverse samples and controlled designs will be critical to confirm these findings and to guide the integration of REN into pediatric headache management pathways.

## 5. Conclusions

In this preliminary real-world study, the Nerivio REN device demonstrated meaningful clinical benefits for pediatric patients with migraine, with primary outcomes showing headache improvement and exploratory measures suggesting potential functional gains. Most patients expressed satisfaction, and more than 80% reported a willingness to continue use, underscoring the device’s acceptability in routine care. Although these findings are based on retrospective patient reports from a small, single-site sample, they support the feasibility and potential clinical value of REN as a patient-centered, non-pharmacologic adjunct in pediatric headache management.

## Data Availability

De-identified questionnaire data and the aggregated dataset used for analysis are available from the corresponding author upon reasonable request. Individual patient-level data cannot be shared publicly to protect privacy.
